# Endocarps of menispermaceous plants in Taiwan

**DOI:** 10.1186/s40529-016-0129-7

**Published:** 2016-07-15

**Authors:** Sheng-Zehn Yang, Po-Hao Chen

**Affiliations:** National Pingtung University of Science and Technology, No. 1, Shuefu Rd., Neipu, Pingtung, 912 Taiwan

**Keywords:** Condyle, Classification, Dioecious climbing plants, Mesocarp, Moonseed family

## Abstract

**Background:**

The fruits of the family Menispermaceae are drupes, and because the drupe endocarps are morphologically diverse, they are generally used to classify members of this family. There is a lack of detailed knowledge of Menispermaceae endocarps in Taiwan. Therefore, in this study, the endocarps of seven genera and 14 species were dissected, and their morphological characters were photographed and described. Furthermore, a key for the 14 species based on the endocarps features, as well as a key of the studied genera, is proposed.

**Results:**

The endocarp types comprise a straight shape in *Tinospora dentata* Diels and a horseshoe shape in the other 13 species. In general, the genus *Cyclea* bears two dorsal crests with 16 small spines, although there are about 25 spines in *Cyclea insularis* (Makino) Hatusima. The genus *Cocculus* has a dorsal convex endocarp with a subannular. In Taiwan, the endocarp of *Sinomenium acutum* (Thunb.) Rehder & E. H. Wilson has up to 26 transverse ridges, without spines on the crest and might be categorized under the genus *Menispermum*. Transverse endocarp ridges are found in *Stephania cephalantha* Hayata and *Stephania tetrandra* S. Moore, whereas transverse endocarp strips are found in *Stephania japonica* (Thunb.) Miers, *Stephania longa* Lour. and *Stephania merrillii* Diels.

**Conclusion:**

We believe that the descriptions and photographs of the endocarp traits of *T. dentata*, which is endemic to Taiwan, will provide more evidence for further studies on extant and fossil species.

## Background

The Menispermaceae is a diverse family of mostly dioecious climbing plants, consisting of a few tree species, but mostly shrubs, and herbs. There are 72 genera and 520 species in this family (Jacques et al. 2011; Wang et al. 2012), and the most speciose genera include *Cissampelos* L. (20–30 spp.), *Cyclea* Arn. ex Wight (30 spp.), *Stephania* Lour. (30–60 spp.), and *Tinospora* Miers (25–35 spp.) (Wefferling et al. 2013). Most members of this family are distributed throughout the tropics, although some are found in temperate regions. Their endocarps appear often in fossil records, and they indicate the presence of a wet forest ecosystem (Herrera et al. 2011). About 116 species in this family were surveyed for their endocarp traits by Jacques (2009).

Menispermaceae leaves are typically simple, with a palmatinerved venation, although a few genera have a pinnate venation. Their leaves are subpeltate, with a few taxa’s leaves being strongly peltate, with pulvinate petioles at both ends. Menispermaceae inflorescences and infructescences are mostly determinate; axillary, or borne on defoliate branches or old wood, rarely terminal; solitary or fasciculate; occur most often in racemes, cymes, or thyrses, sometimes in panicles or cymose heads, rarely reduced to solitary flowers (Wefferling et al. 2013). Flowers are unisexual, inconspicuous, trimerous, and actinomorphic; a few genera have pistillate, zygomorphic flowers; carpels are free, usually range between three and six in number, whereas the zygomorphic genera only have a single carpel; stamens are mainly free, but some genera form a synandrium (Ortiz et al. 2007). Fruits are produced aggregates of 3 or 6, single-seeded, and sometimes stipitate drupes. They usually consist of a fleshy or fibrous mesocarp, and a bony, woody, coriaceous, chartaceous or papyraceous endocarp.

The dorsal portion of the fruit in this family is mostly convex. Otherwise, it grows in a gibbous manner that curves the fruit into a horseshoe or crescent-shape, with the style scar becoming basal or sub-basal, appearing in mature fruit close to the pedicel. Otherwise, if the fruit is not curved, the style scar may be apical. Many endocarp characters vary within the family, such as endocarp type, limb length, condyle shape, convex or concave dorsal face, chambers, vascular trace, aperture, and perforation. Because this family is mainly defined by the curved seed found in many of the genera, it is also known as the moonseed family.

Menispermaceae endocarps vary widely in their ornamentation, which provides important taxonomic characters for distinguishing between and within the genera. Six genera and 13 taxa (including one variety) of this family have been identified in the Flora of Taiwan (Huang and Huang 1996), but exhaustive information on Menispermaceae endocarps is still lacking. This lack of data is addressed in this study, using their diverse endocarp ornamentation, by (1) carefully revising the various endocarp descriptors and providing detailed photographs of the discussed features, (2) developing a key based on endocarp morphological characters to identify the Menispermaceae genera and species in Taiwan.

## Methods

To fully examine the endocarp morphology of the taxa, and in preparation for their photography, we collected fresh fruits of 14 species in seven genera belonging to Menispermaceae in Taiwan from 2012 to 2015 (species listed in Appendix 1). Fruits were studied using material obtained from the Herbarium of Taiwan Forestry Research Institute (TAIF), the Provincial Pingtung Institute (PPI), or personal collections.

We prepared the endocarp material after Forman (1986), Tiffney (1991), Jacques (2009b), and Herrera et al. (2011). First, the fruits were hydrated in boiling water for about 10 min, and subsequently left in water overnight to soften the pericarp. Second, the pericarp and mesocarp were separated using fine pliers, a small sharp scalpel, or a nylon toothbrush, and subsequently rinsed until free of soft tissues. The soft tissue adhering to the surface of the endocarp was brushed away as much as possible, and then cleaned with the toothbrush. When hydrated, spines of some endocarps are only slightly flexible and easily broken when dried, and must therefore be handled carefully. Mesocarp tissue was removed after hydration with boiling water, or by soaking the fruits in tap water, depending on their size and mesocarp thickness. The adhesion of the mesocarp to the endocarp is highly variable, even intraspecifically, such that this variability is linked to differences in species, maturity, and drying process. Finally, the endocarps were left in petri dishes to air-dry for at least 1 day.

Photographs of the endocarps were taken from lateral view, dorsal view, and sagittal view using a Nikon D80 SLR digital camera (Lens AF Micro Nikkor 60 mm 1: 2.8D, Nikon Corporation, Tokyo, Japan), in the herbarium of PPI. The endocarp dimensions were measured using Image-J software (Ferreira and Rasband 2011), and if multiple specimens were examined, the average size was recorded. Furthermore, data were supplemented with available images and descriptions from other published studies (Thanikaimoni 1986; Ortiz et al. 2007; Jacques 2009a; Liu Jacques 2010; Herrera et al. 2011; Wefferling et al. 2013; Ortiz and Nee 2014). The terminology for the description of the endocarps partly follows Jacques (2009a), and a complete definition of endocarp characters can be found in Appendix 2.

Overall, we examined the endocarp variation among 14 species in the family Menispermaceae. The endocarp measurements and descriptions included four parts: (1) endocarp type, surface type, length (mm), width (mm), and thickness (mm); (2) lateral view, including the rows of ridges, number of transverse ridges or strips, number of spines, perforation, aperture, chambers, vascular trace, condyle shape, limb length; (3) dorsal view, including the rows of ridges, number of spines, number of transverse ridges, groove presence; (4) ventral view, including ventral trace, longitudinal aperture, keeled at apex.

## Results

The morphological descriptions of some species’ endocarps have been reported in earlier studies. However, for all 14 investigated species the photographs and observations are new. The endocarp descriptions of these 14 investigated species were used to construct a comparison table. The identifying details, including the endocarp type; the surface type; and a lateral, dorsal, and ventral view, appear in Table 1, as well as in figures as follows: *Cissampelos pareira* L. var. *hirsuta* (DC.) Forman (Figs. 1–5), *Cocculus laurifolius* DC. (Figs. 6–9), *Cocculus orbiculatus* (L.) DC. (Figs. 10–13), *Cyclea gracillima* Diels (Figs. 14–18), *Cyclea insularis* (Makino) Hatusima (Figs. 19–23), *Cyclea ochiaiana* (Yamam.) S. F. Huang & T. C. Huang (Figs. 24–28), *Pericampylus glaucus* (Lam.) Merr. (Figs. 29–33), *Sinomenium acutum* (Thunb.) Rehder & E. H. Wilson (Figs. 34–37), *Stephania cephalantha* Hayata (Figs. 38–43), *Stephania japonica* (Thunb.) Miers. (Figs. 44–48), *Stephania longa* Lour. (Figs. 49–54), *Stephania merrillii* Diels (Figs. 55–59), *Stephania tetrandra* S. Moore (Figs. 60–64), *Tinospora dentata* Diels (Figs. 65–69). Three views are shown for all figures, and each figure is indicated using abbreviated labels to show the relevant parts of the endocarp.

**Table 1:** Morphological characteristics of endocarps and flower parts in Menispermaceae in Taiwan

Characters	*Cissampelos pareira* L. var. *hirsuta* (DC.) Forman	*Cocculus laurifolius* DC.	*Cocculus orbiculatus* (L.) DC.	*Cyclea gracillima* Diels	*Cyclea insularis* (Makino) Hatusima	*Cyclea ochiaiana* (Yamam.) S. F. Huang & T. C. Huang	*Pericampylus glaucus* (Lam.) Merr.	*Sinomenium acutum* (Thunb.) Rehder & E. H. Wilson	*Stephania cephalantha* Hayata	*Stephania japonica* (Thunb.) Miers	*Stephania longa* Lour.	*Stephania merrillii* Diels	*Stephania tetrandra* S. Moore	*Tinospora dentata* Diels
Types	Horseshoe	Horseshoe	Horseshoe	Horseshoe	Horseshoe	Horseshoe	Horseshoe	Horseshoe	Horseshoe	Horseshoe	Horseshoe	Horseshoe	Horseshoe	Straight
Surface	Rugose	Reticulated	Reticulated	Scabrid	Scabrid	Scabrid	Scabrid	Short ridges	Excavated	excavated	Excavated	Excavated	Excavated	Scabrid
Length (mm)	3.4–3.5	4.3–5.2	3.1–3.8	2.7–2.9	4.2–4.8	4.1–4.2	4.5–6.3	5.1–5.5	4.7–5.7	5.0–5.3	5.8–6.5	9.1–10.3	4.8–5.0	8.0–8.8
Width (mm)	3.4–3.5	4.6–5.3	3.4–4.0	2.7–2.8	3.9–4.4	3.9–4.1	4.5–5.7	6.7–7.2	4.4–5.7	4.8–5.2	4.9–5.4	8.5–9.2	4.3–4.5	6.9–7.2
Thick (mm)	1.8–1.9	3.6–4.2	2.2–2.5	2.1–2.2	2.2–2.7	1.8–2.2	2.1–2.3	2.1–2.3	2.4–2.9	2.2–2.5	2.3–2.7	3.3–4.5	1.7–1.9	5.7–5.9
*Lateral view*														
Row of ridges	1	1	1	2	2	2	2	1	1	1	1	1	1	2–3
Number of transverse ridges	Obscure	Obscure	9	–	–	–	–	30	12	–	–	–	17	–
Number of transverse strips	–	–	–	–	–	–	–	–	–	9	10	14	–	–
Number of spines	–	–		15, 17	15, 23	13, 13	15, 20	–	–	–	–	–	–	Obscure
Perforated	1	0	0	0	0	0	0	0	0/1	1	0/1	1	0	0
Chamber	0	1	1	1	1	1	0	0	0	0	0	0	0	0
Vascular trace	0	0	0	0	0	0	0	1	0	0	0	0	0	0
Aperture	0	0	0	1	1	1	0	0	0	0	0	0	0	0
Condyle	0	0	0	0	0	0	0	0	0	0	0	0	0	1
Limbs	0	1	0	0	1	0	1	0	0	0	1	1	1	0
*Dorsal view*														
Row of ridges	2	2	2	2	2	2	2	1	2	2	2	2	2	1
Number of spines	–	–	–	17, 17	25, 25	17, 17	20, 20	27	–	–	–	–	–	Numerous
Number of transverse ridges	7, 7	–	–	–	–	–	–	–	12, 12	–	–	–	17, 17	–
Number of transverse strips	–	–	–	–	–	–	–	–	–	9, 10	10, 11	12, 12	–	–
Groove	1	1	1	0	0	0	0	1	1	0	0	0	0	0
*Ventral view*														
Ventral trace	1	1	1	1	1	1	1	0	1	1	1	1	1	0
Longitudinal aperture	0	0	0	0	0	0	0	0	0	0	0	0	0	1
Keeled at apex	0	0	0	0	0	0	0	0	0	0	0	0	0	1
*Flower parts*														
Androecium	0	1	1	0	0	0	1	1	0	0	0	0	0	1
Style position	0	0	0	0	0	0	0	0	0	0	0	0	0	1

*0* absent, *1* present, *0/1* absent/present; *longitudinal aperture*: *0* absent, *1* present; *chamber* 0 absent, *1* present; *vascular trace*: *0* absent, *1* present; *condyle*: *0* compressed, *1* chamber; *limb*: *0* equal in length, *1* unequal in length; groove: *0* absent, *1* present; *dorsal convex*: *0* absent, *1* present; *keeled at apex*: *0* absent, *1* present; *androecium*: *0* synandrium, *1* no synandrium; *style position*: *0* basal, *1* terminal

**Figs. 1–18:**
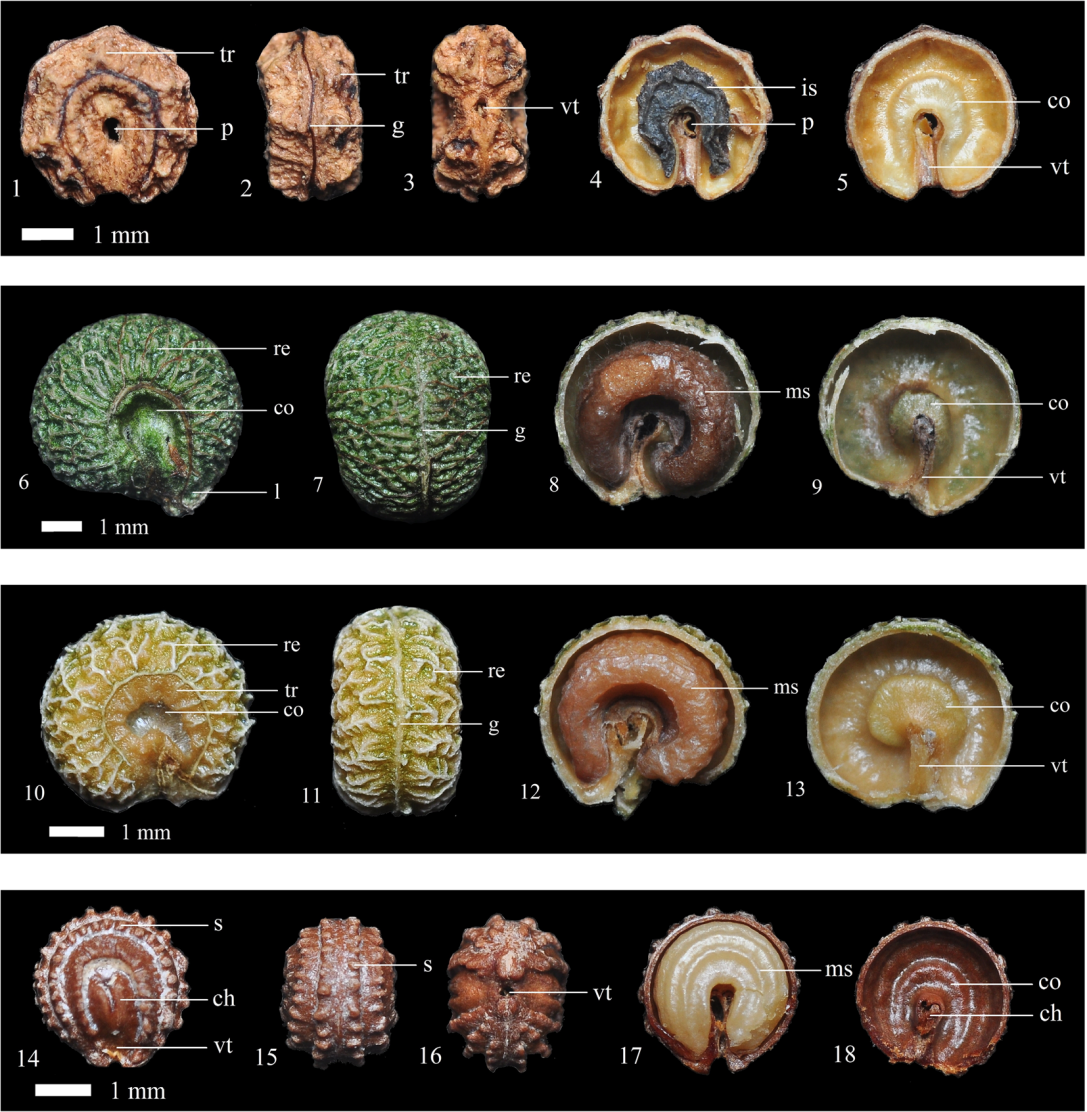
Menispermaceae endocarps. **1–5** *Cissampelos pareira* L. var. *hirsuta* (DC.) Forman (PPI, P. H. Chen 502). **1** Lateral view, obscure transverse ridges with a prominent perforation. **2** Dorsal view, a groove. **3** Ventral view, vascular trace. **4** Sagittal view, immature seed. **5** Sagittal view empty, inner endocarp. **6–9**. *Cocculus laurifolius* DC. (PPI, P. H. Chen 964). **6** Lateral view, unequal length of limbs. **7** Dorsal view, conspicuous dorsal convex, surface with reticulated ridges. **8** Sagittal view, seed. **9** Sagittal view empty, inner endocarp. **10**–**13** *Cocculus orbiculatus* (L.) DC. (PPI, P. H. Chen 769). **10** Lateral view, same length of limbs. **11** Dorsal view, surface with reticulated ridges. **12** Sagittal view, seed. **13** Sagittal view empty, inner endocarp. **14**–**18** *Cyclea gracillima* Diels (PPI, P. H. Chen 564). **14** Lateral view, two lateral ridges with conspicuous chambers and spines. **15** Dorsal view, two dorsal ridges, dorsal convex. **16** Ventral view, vascular trace. **17** Sagittal view, seed, and same length of limbs. **18** Sagittal view empty, inner endocarp. *ch* chamber, *co* condyle, *g* groove, *is* immature seed, *ms* mature seed, *l* limb, *p* perforated, *re* reticulated, *s* spine, *tr* transverse ridges, *vt* vascular trace

**Figs. 19–37:**
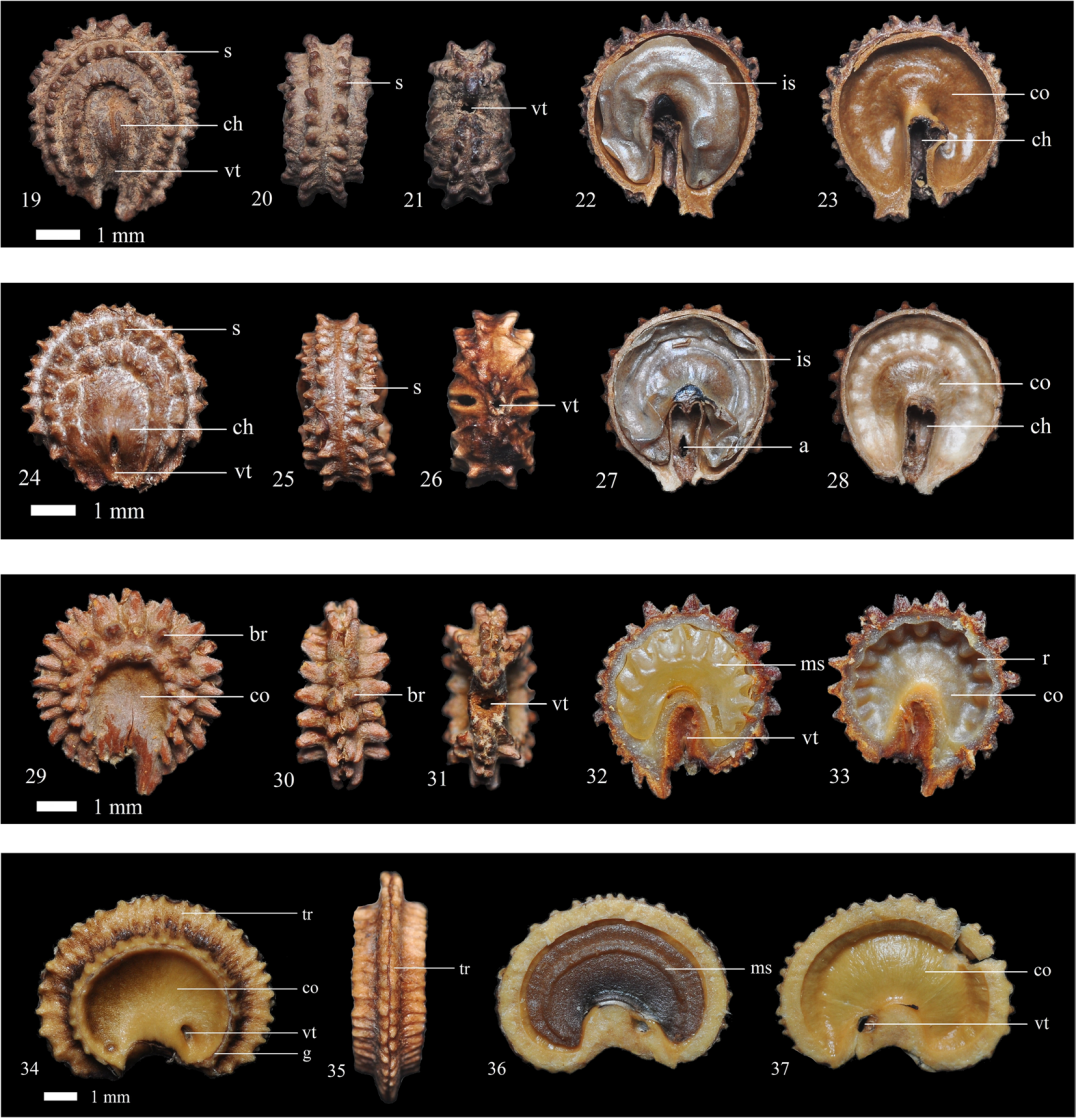
Menispermaceae endocarps. **19**–**23** *Cyclea insularis* (Makino) Hatusima (TAIF, Chiu & Chen 4116). **19** Lateral view, two lateral ridges with outer wall of chambers and spines. **20** Dorsal view, two dorsal ridges with spines. **21** Ventral view, vascular trace. **22** Sagittal view, seed, showing unequal length of limbs. **23** Sagittal view empty, inner endocarp. **24**–**28** *Cyclea ochiaiana* (Yamam.) S. F. Huang & T. C. Huang (PPI, P. H. Chen 513). **24** Lateral view, conspicuous chambers and spines. **25** Dorsal view, two dorsal ridges with spines. **26** Ventral view, vascular trace. **27** Sagittal view, seeds, showing unequal length of limbs. **28** Sagittal view empty, inner endocarp. **29**–**33** *Pericampylus glaucus* (Lam.) Merr. (PPI, P. H. Chen 430). **29** Lateral view, broad dorsal crests. **30** Dorsal view, conspicuous spines, **31** Ventral view, vascular trace. **32** Sagittal view, seeds exhibits conspicuous ribs, unequal length of limbs. **33** Sagittal view empty, inner endocarp with convex ribs. **34**–**37** *Sinomenium acutum* (Thunb.) Rehder & E. H. Wilson (TAIF, Chung et al. 11713). **34** Lateral view, vascular trace near one limb and a vascular notch, number of short transverse ridges (protuberance) >26. **35** Dorsal view, number of dorsal ridges >26. **36** Sagittal view, seed crescent–shaped with ribs. **37** Sagittal view empty, entry of lateral vascular tube. *a* aperture, *br* broad ridge, *ch* chamber, *co* condyle, *g* groove, *is* immature seed, *ms* mature seed, *p* perforated, *r* ribs, *s* spine, *tr* transverse ridges, *vt* vascular trace

**Figs. 38–59:**
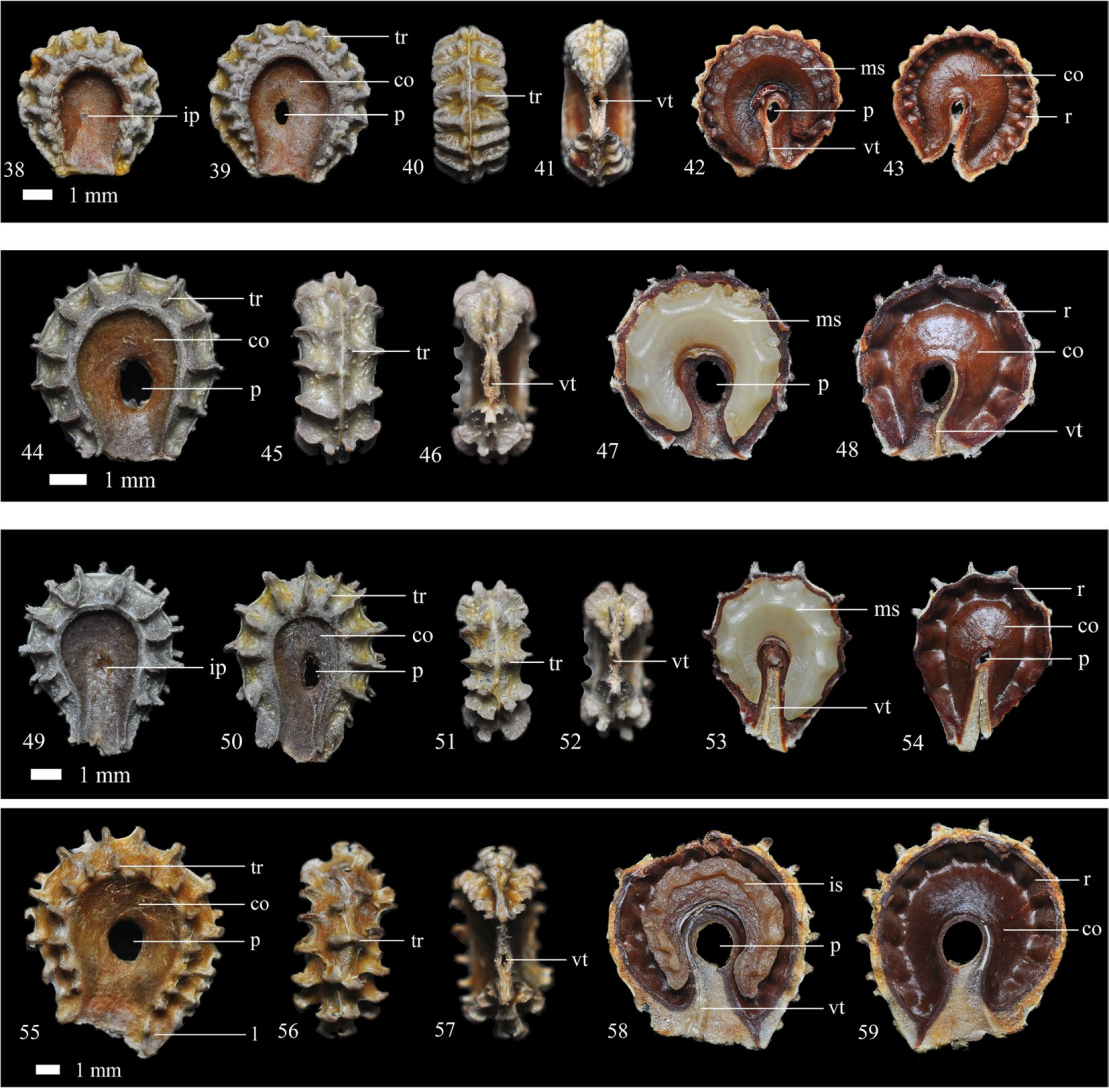
Menispermaceae endocarps. **38**–**43** *Stephania cephalantha* Hayata (PPI, P. H. Chen 860). **38** Lateral view, incomplete perforation. **39** Lateral view, a prominent perforation. **40** Dorsal view, transverse ridges. **41** Ventral view, vascular trace. **42** Sagittal view, seed and unequal length of limbs. **43** Sagittal view empty, intrusive ribs in locule and course of straight vascular trace through the solid condyle. **44**–**48** *Stephania japonica* (Thunb.) Miers (PPI, P. H. Chen 416). **44** Lateral view, shape ovate with a prominent perforation. **45** Dorsal view, transverse ridges (strips). **46** Ventral view, vascular trace. **47** Sagittal view, seed and unequal length of limbs. **48** Sagittal view empty, intrusive ribs in locule and course of straight vascular trace through the solid condyle. **49**–**54** *Stephania longa* Lour. (PPI, P. H. Chen 792). **49** Lateral view, incomplete perforation. **50** Lateral view, a prominent perforation. **51**. Dorsal view, transverse ridges (strips).**52** Ventral view, vascular trace. **53**. Sagittal view, seed and unequal length of limbs. **54** Sagittal view empty, intrusive ribs in locule and course of straight vascular trace through the solid condyle. **55**–**59** *Stephania merrillii* Diels (PPI, P. H. Chen 150). **55** Lateral view, a prominent perforation. **56** Dorsal view, transverse ridges (strips). **57** Ventral view, vascular trace. **58** Sagittal view, seed and longer seed limb. **59** Sagittal view empty, intrusive ribs in locule and course of straight vascular trace through the solid condyle. *co* condyle, *is* immature seed, *ip* incomplete perforation, *l* limb, *ms* mature seed, *r* ribs, *p* perforated, *tr* transverse ridges, *vt* vascular trace

**Figs. 60–69:**
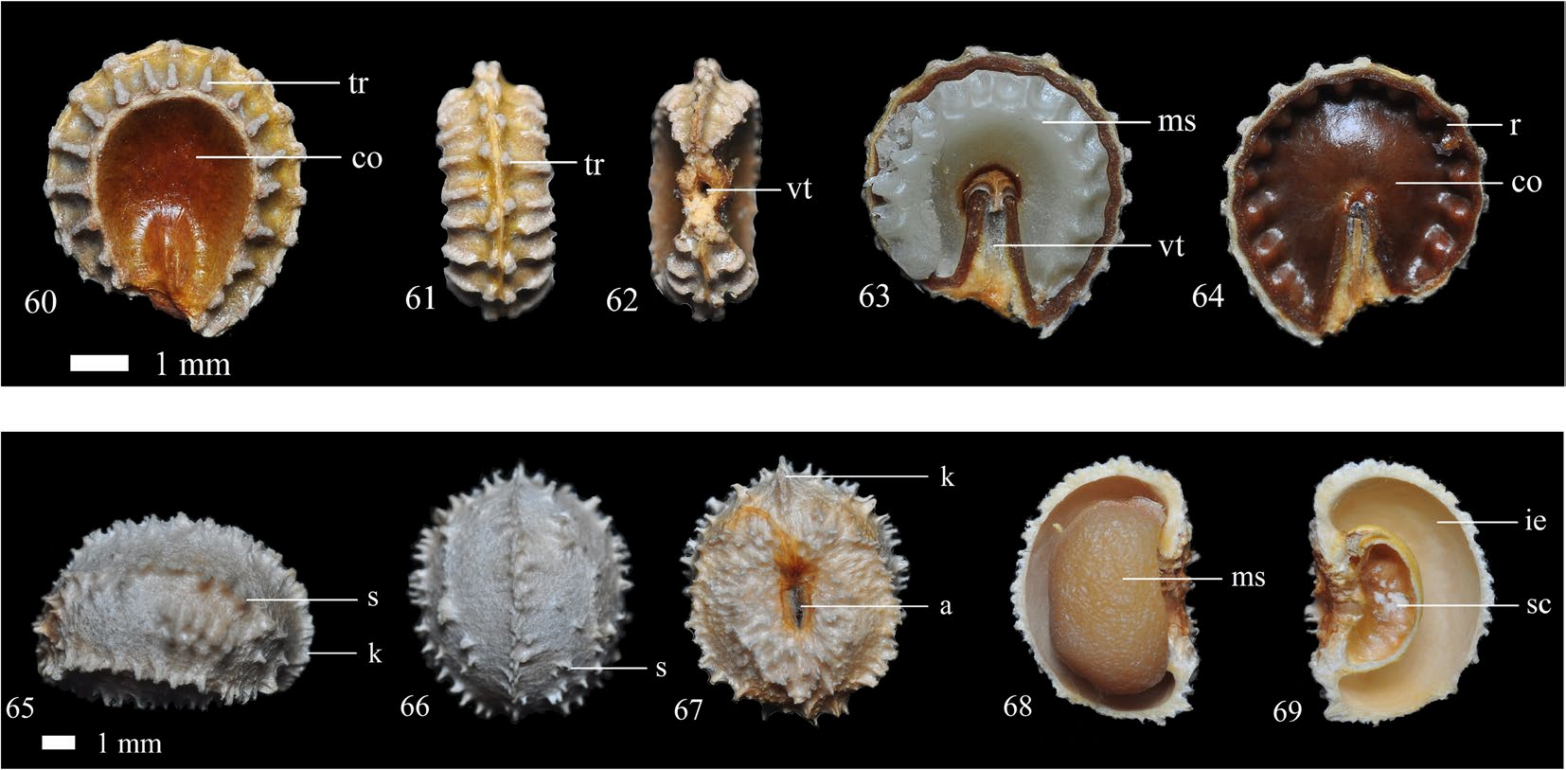
Menispermaceae endocarps. **60**–**64** *Stephania tetrandra* S. Moore (PPI, P. H. Chen 415). **60** Lateral view, incomplete perforation with convex sculpturing near ventral notch. **61** Dorsal view, transverse ridges. **62** Ventral view, vascular trace. **63** Sagittal view, seed and unequal length of limbs. **64** Sagittal view empty, straight vascular tube. **65**–**69** *Tinospora dentata* Diels (PPI, P. H. Chen 361). **65** Lateral view, two or three slightly lateral ridges with spines on the surface. **66** Dorsal view, one dorsal ridge. **67** Ventral view, a longitudinal aperture of linear condyle, keeled at apex. **68** Sagittal view, seed. **69** Sagittal view empty, empty endocarp, showing subglobose condyle. *a* aperture, *co* condyle, *i.e.* inner endocarp, *k* keeled, *ms* mature seed, *r* ribs, *s* spine, *sc* subglobose condyle, *tr* transverse ridges, *vt* vascular trace

The endocarps of 14 menispermaceous species in Taiwan displayed both horseshoe and straight types, and their length ranged from 2.1 to 10.3 mm (Table 1). At the generic level, their shape was generally similar, but their ornamentation varied among species. The dorsal ridges were more or less symmetric, and the lateral faces did not have a convex sculpturing in the genera *Cissampelos, Cyclea,* and *Stephania.* Based on these endocarp characteristics, we constructed a key to seven genera (Appendix 3), based on the Menispermaceae fruit by Wefferling, et al. (2013). We furthermore established a key to the 14 species, allowing identification to the level of genera and species, as follows: .

## Discussion

The family Menispermaceae is divided in two large clades, with the tribes Tinosporeae and Menispermeae included in clades 1 and 2, respectively. Molecular phylogeny was studied using five characteristics either from the flower, i.e., using the androecium and style scar, or from the seed, i.e., using the endosperm, cotyledons, and shape (Ortiz et al. 2007; Jacques and Bertolino 2008). According to the previous reports, among the seven genera in this study, the synandrium in the central column with horizontal anthers is a synapomorphy of three genera, i.e., *Cissampelos*, *Cyclea*, and *Stephania*, but in four genera, i.e., *Cocculus*, *Pericampylus*, *Sinomenium*, and *Tinospora*, there are no synandrium. The position of the style scar is terminal in the genus *Tinospora* and basal in the other six genera. Endosperm is present in all seven genera. The cotyledons are foliaceous in *Tinospora* and fleshy in the other six genera. The seed shape is straight in *Tinospora* and crescent in the other genera.

The ancestral conditions for Menispermaceae are synapomorphies of foliaceous cotyledons, no synandrium, and the terminal position of the style scar (Jacques and Bertolino 2008), as well as straight-shaped seeds (Ortiz et al. 2007). In this study, we suggest that the morphological phylogeny of the genus *Tinospora* is the most original condition among seven genera in Taiwan because of foliaceous cotyledons, terminal style scar, and straight-shaped seed; the genera *Cissampelos,* *Cyclea*, and *Stephania* are derived from *Cocculus*, *Pericampylus*, and *Sinomenium* because of a synapomorphy of no synandrium.

The three genera, *Cissampelos, Cyclea,* and *Stephania*, form a clade (*S*–*C*–*C* clade, or pantropical clade in Herrera et al. 2011) that is strongly supported as monophyletic, which has female flowers with a single carpel as a morphological synapomorphy (Wefferling et al. 2013). Furthermore, the endocarps of the three genera are variously ornamented with spines, tubercles, wings, or ridges; distal limb is longer (rarely equal), and sometimes has two (to four) lateral chambers. These chambers can have lateral apertures or not; the vascular trace is ventral; the condyle is compressed bilaterally or both bilaterally and dorsoventrally, and is sometimes perforated (Wefferling et al. 2013).

*Cissampelos* and *Cyclea* are closely related to *Stephania* (Hoot et al. 2009; Jacques et al. 2011), but their endocarps are easily distinguished. In *Cissampelos* and *Cyclea*, there are always two lateral crests (ridges) per side, usually two dorsal crests, and their endocarps are often imperforated. According to published results (Jacques 2009; Liu and Jacques 2010; Herrera et al. 2011), endocarps of *Cissampelos pareira* L. are covered by conspicuous spines with two dorsal and lateral ridges (crests). They also have a distinctly longer distal limb that slightly comes closer, and the outer lateral ridge partly overlaps the inner one (all ridges bear outgrowths). Ortiz and Nee (2014) described a new species of *Cissampelos arenicola* M. Nee & R. Ortiz. Its endocarp length and width are about 6 × 7 mm, and it is suborbicular-bilaterally compressed, with one small circular perforation on the lateral faces. Furthermore, its ornamentation is obscure, consisting of a very low medial ridge and faintly transverse ridges. In this study, the endocarp of *C. pareira* var. *hirsuta* was found to be horseshoe-shaped, with one circular perforation, and one dorsal and lateral ridge (Table 1; Figs. 1–3) that are more or less similar as in *C. arenicola*, but truly differ from those of *C. pareira*. This might be due to intraspecific differences, or because we included immature fruits that had not yet developed those characteristics. However, because of the low number of specimens known for some species (e.g., only two fruits were collected), this study did not consider any intraspecific variability for endocarp shape and size. Therefore, collecting and studying more mature fruits might resolve these inconsistencies in endocarp characteristics.

Forman (1986) and Thanikaimoni (1986) describe in detail that *Cocculus* showed a formation of dome–shaped extensions of the lateral ridges over the median septum that create a two–chambered condyle. The diagnostic characters of *Cocculus* included lateral faces of endocarp without aperture, or with aperture or perforation centered in the lateral concave face. Lateral chambers are present, as well as a surface with longitudinal and transverse ridging. Finally, a lateral compression that is restricted to center of each lateral face results in a subannular endocarp (Wefferling et al. 2013). Furthermore, Jacques (2009b) reported an endocarp morphological description of *C. laurifolius*, *C. orbiculatus*, and those of the genus *Cocculus*. When comparing the figures and terms used in the previous reports for these two species in Taiwan, we observed differences in the characteristics of the subannular shape, the formation of lateral chambers without aperture, a reticulated surface, and their obscure ventral trace (Figs. 6–13), whereas the other characteristics were consistent with the previous results described as above.

Jacques (2009b) included the concave exterior of laterally compressed endocarps as part of the condyle, and referred to a double external condyle in descriptions of several taxa, including *Cissampelos*, *Cocculus*, *Cyclea*, *Pericampylus*, and *Stephania*. In this study, the endocarps of three species, *C. gracillima*, *C. insularis*, and *C. ochiaiana* (Figs. 1–18, 19–28) are consistent with the characteristics described as above, because they have two dorsal and two lateral ridges, and two lateral chambers combined with a condyle aperture on the ventral view. Liu and Jacques (2010) indicated that the genus *Cyclea* bears two dorsal crests with 16 small spines, but there are about 25 spines in *C. insularis*. The latter might be an intraspecific difference or influenced by the measuring method. Wefferling et al. (2013) indicated that the endocarps of the genus *Cyclea* show a variety of ornamentation, with two to four lateral chambers with or without lateral apertures. In the present study, we found that three species in Taiwan display a lateral aperture with different chamber sizes, especially the endemic species *C. insularis* (Figs. 24, 27).

The endocarp of *P. glaucus* shows a lateral concavity (i.e., condyle) a broader dorsal ridge at one limb, a ventral vascular trace, concave lateral faces with convex sculpturing near ventral notch, and long spines or very thick spines (Jacques 2009b; Herrera et al. 2011; Wefferling et al. 2013). The characteristics described above are consistent with the species in Taiwan (Figs. 29–33). The species *Pericampylus formosanus* Diels was placed as the synonym of *P. glaucus* (Lo et al. 2008), but the former can be differentiated from the latter by the covering hairs on both surfaces of the leaf and the filaments united into a tube (Huang and Huang 1996). Future comparative studies on hairs, filaments, and endocarps between these two species might provide strong evidence for the synonym treatment.

The endocarp lateral faces of the genus *Sinomenium* show a conspicuous off-center aperture near distal limb (Wefferling et al. 2013), and *S. acutum* (Herrera et al. 2011) and *S. acutum* var. *cinereum* (Wefferling et al. 2013) have spines on the dorsal ridge. Jacques (2009b) also indicated that the double external condyle of *S. acutum* is not perforated, makes a large central area with a conspicuous hollow near one limb, and has a comma-shaped ridge bordering it. The position of this hollow and the aperture is as same as the one near the limb, but these two researchers describe it by different terms. A personal communication with Dr. Jacques indicated that the aperture must be connected to a subglobose condyle, such as in the genus *Tinospora*. He furthermore suggested that, for the genus *Sinomenium*, it should be called a vascular trace (Table 1). The vascular trace was indeed observed in *S. acutum,* but the spines were not in this study (Figs. 34–37). The key to the genera of Menispermaceae indicated that the number of lateral ridges of *Sinomenium* is <25, and that of *Menispermum* is >26 (Jacques 2009b), and the number of lateral ridges of species in Taiwan is >26. Liu and Jacques (2010) compared fossil endocarps with extant species of *S. acutum* and *Maianthemum canadense* L. In their study, the number of protuberances (i.e., lateral ridges) for *S. acutum* ranged from 18 to 21, and showed ventral notch that was straight or slightly concave, whereas those of *M. canadense* L. ranged between 27 and 30, and the ventral notch was V-shaped. Furthermore, the ornamentation of endocarps of *Menispermum* has no spines on the dorsal crest, the number of lateral ridges is >26, and the ventral notch is almost V-shaped. We therefore question if the species *S. acutum* in Taiwan is misplaced and similar to the *Menispermum* genus. This inconsistency should be addressed with further sampling data.

The endocarp dorsal ridges of the genus *Stephania* are discontinuous, with a strong ornamentation that has an intrageneric variation, namely in the transverse ridges, and in the condyle perforation (Jacques 2009b). In this study, the genus *Stephania* displays the crustaceous, transverse ridging that is different from *Cissampelos* and *Cyclea*. Transverse ridges of endocarps are found in *S. cephalantha*, and *S. tetrandra* (Figs. 38–43, 60–64), whereas transverse strips are found in *S. japonica*, *S. longa* and *S. merrillii* (Figs. 44–59). The other lateral face characters in this genus are consistent with the previous results (see Table 1). Concave lateral faces are present, and have a convex sculpturing near the ventral notch in *S. tetrandra* (Fig. 60). This character is only found in the genus *Pericampylus* (Wefferling et al. 2013).

The endocarp of the genus *Tinospora* is bony, round, ovoid/obovoid, or subellipsoidal in outline, tuberculate or verrucose, sometimes echinate, hispid (with stiff bristles), or ornamented with wings and ridges. The lateral chambers are absent, the vascular trace is positioned inside the ventral concavity of the condyle and forms a cylindrical, protruding vascular tube extends nearly to the level of the aperture mouth; the condyle is hollow, deeply intrusive, and subglobose or subreniform, and it is open ventrally (Wefferling et al. 2013). In the present study, the endocarp of *T. dentata*, which is an endemic species in Taiwan (Hengchun Peninsula), is round in shape with a developed keel at the apex on the dorsal ridge, and a linear condyle aperture; the condyle is subglobose in shape (Fig. 69), with a cupule and locule, and a protruding vascular tube. Lo et al. (2008) did not report this drupe information, and this is the first description and photographs for the endocarp of *T. dentata* (Table 1; Figs. 65–69) providing some evidence and references for further studies.

Birds and mammals are the most commonly observed fruit consumers in Menispermaceae. Birds species known disperse fruits and seeds include the eastern phoebe flycatchers (dispersing *Cocculus* DC.; Kessler 1993). Mammalian dispersal vectors include gorillas, lemurs, galagos (dispersing *Cissampelos*; Jacques 2009b), and white-faced monkeys (dispersing *Cissampelos*; Croat 1978). Forman (1986) points to humans as a recent and significant dispersal agent. Relatively little is known about menispermaceous fruit dispersal or seed predation within the family. Furthermore, the knowledge of endocarp adaptation is limited by the lack of information on the seed dispersal. Therefore, ecological studies and understanding of endocarp dispersal should provide new insights in the adaptive role of some endocarp features.

The molecular phylogeny of the three species *C. orbiculatus, P. glaucus,* and *S. japonica,* collected from Taiwan and preserved the herbarium (MO and HAST), has been reported (Ortiz et al. 2007). That study emphasized the importance of specimen exchanges among herbaria for different field studies. Some inconsistencies in our results could be addressed with an extensive sampling of fruits and genomic sequencing of the data. The endocarp characters we described can provide some phylogenetic information for a morphological phylogenetic analysis of this family.

## Conclusion

The synapomorphies of the androecium, style scar, seed shape, and cotyledons for the family Menispermaceae are supported for morphological phylogeny. The genus *Tinospora* may be the most original condition among seven genera of Taiwan with its characteristics of terminal style position, straight-shaped seed, and foliaceous cotyledons. The three species *C. ochiaiana,* *S. merrillii,* and *T. dentata,* are endemic for Taiwan, and their endocarp information will provide guidelines for the identification or phylogenetic analysis of this family. The endocarp of *C. pareira* var. *hirsuta* is significantly different from that of *C. pareira* and should be further investigated. Surprisingly, the two dorsal ridges with 25 spines in *C. insularis* differ significantly from previous reports that the genus *Cyclea* has about 16 small spines. The endocarp of *Cocculus* is subannular in shape, with a dorsal convex face. The different characteristics between the genera *Sinomenium* and *Menispermum* will be useful for taxonomic determinations. Transverse ridges and strips on the dorsal and lateral face of endocarps are found in the genus *Stephania.* Finally, we provide the first description and photographs of endocarp characteristics for *T. dentata.* This information will provide a reference for further studies.

## Appendix 1

See Table 2.

**Table 2:** Species of Menispermaceae photographed and described for endocarp morphology

Scientific name	Herbarium, number	Regions	Altitudes (m)
*Cissampelos pareira* L. var. *hirsuta* (Buch. ex DC.) Forman	PPI, 76635	Hsiaoliouchiou Island, Pingtung County, southern Taiwan	20–30
*Cocculus laurifolius* DC.	PPI, 76899	Taiwu, Pingtung County, southern Taiwan	400–600
*Cocculus orbiculatus* (L.) DC.	PPI, 76884	Machia, Pingtung County, southern Taiwan	600–800
*Cyclea gracillima* Diels	PPI, 76851	Chiyenshan, Pingtung County, southern Taiwan	700–800
*Cyclea insularis* (Makino) Hatusima	TAIF, 199368	Lanyu, Taitung County, southern Taiwan	10–20
*Cyclea ochiaiana* (Yamam.) S. F. Huang & T. C. Huang	PPI, 76742	Peitawushan, Pingtung County, southern Taiwan	1600–1800
*Pericampylus glaucus* (Lam.) Merr.	PPI, 76111	Liangshan, Pingtung County, southern Taiwan	200–300
*Sinomenium acutum* (Thunb.) Rehder & E. H. Wils.	TAIF, 460257	Siyuan Wind Gap, Yilan County, northern Taiwan	1900–2000
*Stephania cephalantha* Hayata	PPI, 76935	Majia, Pingtung County, southern Taiwan	600–800
*Stephania japonica* (Thunb.) Miers	PPI, 76121	Taiwu, Pingtung County, southern Taiwan	600–800
*Stephania longa* Lour.	PPI, 76907	Liangshan, Pingtung County, southern Taiwan	100–200
*Stephania merrillii* Diels	PPI, 75719	Lanyu, Taitung County, southern Taiwan	200–300
*Stephania tetrandra* S. Moore	PPI, 76089	Majia, Pingtung County, southern Taiwan	30–50
*Tinospora dentata* Diels	PPI, 76066	Shihkechienshan, Pingtung County, southern Taiwan	700–800

## Appendix 2: List of morphological characters and definition of endocarps

1. Apertures: various shape, provide a window to the lateral chambers from the lateral faces.
2. Bilateral and dorsoventral curvature: endocarps with a subhemispherical ventral invagination or condyle.
3. Bilateral curvature: endocarps with a longitudinal ventral groove corresponding with the condyle.
4. Condyle: three interpretations: (1) condyle is a placentary process, including middle and inner regions of the ovary wall (for convex condyles) or primarily inner regions of the ovary wall (for bilaterally compressed condyles) in condyle development. (2) condyle is a ventral intrusion of the endocarp. This intrusion may form a hollow cavity or chamber. (3) concave exterior of laterally compressed endocarps is part of the condyle. In other words, condyle denotes the depressed central part, which is delimited by lateral crest and ventral notch. Condyle is the result of an enlarged and protruding placenta or ovary wall, driving the locule to the dorsal and sometimes lateral portions of the endocarp, manifested in the mature fruit as a ventral intrusion (or pair of intrusions) of the endocarp wall into the locule, excluding the seed coat.
5. Dorsoventral curvature: results in hippocrepiform or crescent-shaped endocarps.
6. Endocarp curvature: occurred in several dimensions, bilaterally, dorsoventrally, or both.
7. Endocarp surfaces: covered by tuberculate, scabrid, reticulate, or rugose, described by different terms, such as transverse and reticulated low ridges, longitudinal and transverse ridging, tuberculate, scabrid, reticulate, or rugose.
8. Groove: a linear depression at the surface of endocarp wall.
9. Horseshoe-shaped endocarp: curved dorsoventrally.
10. Lateral chamber: a cavity enclosed or nearly enclosed within the endocarp but distinct from the condyle.
11. Length: represents the longer one and width its perpendicular.
12. Limbs: apical or basal part of a curved endocarp. When only dorsoventral curvature takes place, the two limbs created can vary. One limb is often longer than the other; they may both curve at the extremity, or one limb may have a broader dorsal ridge. Sometimes, the two limbs are more or less equal in length and parallel.
13. Perforated: endocarps may also be perforate, that it is thought to develop from a globular region of cells in the developing fruit dying and leaving a hollow space. This perforation may be complete, or incomplete.
14. Ridging: be longitudinal, transverse, or both, or manifested as spines, or wings (flattened extensions of the endocarp wall).
15. Straight endocarp: endocarp not curved.
16. Thickness: measured perpendicularly to the longitudinal plane of symmetry in case of curved endocarp, but on it and perpendicular to base-apex line in case of straight endocarp.
