# Erratum to: Endocarps of menispermaceous plants in Taiwan

**DOI:** 10.1186/s40529-017-0174-x

**Published:** 2017-04-06

**Authors:** Sheng‑Zehn Yang, Po-Hao Chen

**Affiliations:** National Pingtung University of Science and Technology, No. 1, Shuefu Rd., Neipu, Pingtung, 912 Taiwan

## Erratum to: Bot Stud (2016) 57:14 DOI 10.1186/s40529-016-0129-7

In our recent article (Yang and Chen 2016), we regret that we did not acknowledge that Appendix 2 and Appendix 3 were adapted from Wefferling et al. (2013). In addition, some sentences in the Introduction and Discussion of our article were taken from Wefferling et al. (2013) to describe the menispermaceous endocarps that we studied in Taiwan. We should have ensured transparency in our article as we did not intend to attribute the prior work of Wefferling et al. to ourselves. We apologize to Wefferling et al. for the lack of appropriate attribution.
